# Social Robotics and Dementia: Results from the eWare Project in Supporting Older People and Their Informal Caregivers

**DOI:** 10.3390/ijerph192013334

**Published:** 2022-10-16

**Authors:** Giulio Amabili, Giacomo Cucchieri, Arianna Margaritini, Marco Benadduci, Federico Barbarossa, Riccardo Luzi, Giovanni Renato Riccardi, Giuseppe Pelliccioni, Elvira Maranesi, Roberta Bevilacqua

**Affiliations:** 1 Scientific Direction, IRCCS INRCA, 60124 Ancona, Italy; 2 Medical Direction, IRCCS INRCA, 60127 Ancona, Italy; 3 Clinical Unit of Physical Rehabilitation, IRCCS INRCA, 60127 Ancona, Italy; 4 Unit of Neurology, IRCCS INRCA, 60127 Ancona, Italy

**Keywords:** older people, dementia, cognitive decline, social robotics, informal caregivers, HRI

## Abstract

The aim of this paper is to describe the results collected with the Italian study conducted within eWare project, aimed at supporting the autonomy and health of the older people affected by dementia and their informal caregivers, through the use of an innovative system based on a social robot and a sensorized environmental infrastructure. Nine dyads of older participants with their caregivers were enrolled in testing the system for 6 months. The results show a positive impact of the system in supporting the achievement of personal goals of the participants, as well as in supporting the quality of life of the informal caregivers. Nevertheless, the impact of the system in reducing the caregivers’ burden needs to be deeply investigated. This research highlights the potential of the eWare system but modifications will have to be made, especially on the interactivity capabilities, in order to meet the peculiar needs and wishes of older people with dementia and to favor the long-term use of the system.

## 1. Introduction

At the present, millions of people are affected by dementia (PwD) worldwide, and about 60 to 70 percent of them are diagnosed with Alzheimer’s disease [1]. There are 50 million cases of Alzheimer’s disease and by 2050 this number is thought to triple [2]. It is known that PwD often rely heavily on families and close others for support [3]. In fact, due to the increasing costs in health care and changes in laws and legislations, the family of a person with dementia is increasingly responsible for the care of older people who are no longer self-sufficient. Some studies conducted in Italy confirmed that the management of dementia patients places a particular burden on the caregiver and involves several economic and social costs [4]. Due to the ongoing of the disease, the burden increases, since the worsening of cognitive condition and the decreased autonomy in daily activities may cause distress, anxiety, depression, and impaired physical health [4]. Moreover, unfulfilling needs of PwD is related to a greater risk of nursing home admission and death [5,6]. All those facts show that we are facing a challenge in ensuring care and quality of life for people with dementia.

In order to prolong the time period when the informal caregiver can provide care and support to PwD, it is necessary to reduce their distress. For this purpose, among all explored solutions [7], lifestyle monitoring is considered effective [8,9]. Lifestyle monitoring provides information about daily activities of a person with dementia. The collection of insights for both the short and long-term patterns of life, allows the caregiver to know needs, and in turn how to improve care. Moreover, social robotics can contribute positively to older people’s wellbeing, by allowing people with dementia to live at home and thus improving their perceived quality of life [10,11]. Numerous studies, in fact, have stressed the relevance of including social robots as a complementary strategy to assist older people with dementia in daily activities and to partially relieve the informal caregivers’ stress, for example providing repetitive tasks, reminders and surveillance [11,12,13,14]. Nevertheless, studies on PARO robot suggest that stimulating older people with dementia by giving different feedbacks at visual, hearing and tactile level, may strongly support them in engaging with caregivers, even if unspecific tasks are provided by the robot [15]. Thus, among the most relevant questions in case of dementia, what to favor the most between robotic platforms with enhanced Human–Robot Interaction (HRI) capabilities or easy-to-use service robots that cooperate for specific tasks still remains debated [16,17].

In this paper, the eWare ecosystem, developed inside the AAL project (AAL-2016-071), is presented along with the results gathered during six months’ experimentation in Italy (https://www.aal-europe.eu/projects/eware/, accessed on 2 August 2022).

The overall aims of eWare are to reduce subjective stress of both the informal caregivers and the patient community, to enhance quality of life of people with dementia and their informal caregivers, as well as supporting communication between formal and informal caregivers. These goals should be reached by early warning for crises followed by more in-time interventions and a general reassurance by the ability to monitor the current situation of the person with dementia. Furthermore, eWare aims to support people with dementia to feel more confident and more positive in dignity through meaningful interactions and reminders via a personalized friendly robot.

The eWare eco-system is composed of two main technologies: the Sensara, a lifestyle-monitoring technology, and Tinybot, a social robot. The Sensara consists of few motion (PIR) and open/close sensors (5 to 8 depending on the house), that were installed at strategic places in the older adults’ home (e.g., living room, bathroom, kitchen, hallway and front door) and wirelessly connected to a gateway. Data collected by Sensara were uploaded to an analytics engine in the cloud that, after two weeks of acquisition, was able to recognize living patterns of the person at home: at what time the person gets up, how long the bathroom is used, how often the kitchen is used, how long one is out of the house, when the person is taking a nap. Once the behavioral patterns are known, deviations from habitual patterns could be detected. In case of such deviations or downward trends, the system sent a notification to the informal caregiver.

The Tinybot provided stimulus for individuals with dementia by talking, giving friendly suggestions, reminders, and playing personal music. Additionally, the Tinybot’ artificial intelligence (AI) learned over time and adapted to support the individuals’ specific needs. Both formal and informal caregivers could personalize the Tinybot through an app, by adding their own reminders, suggestions, and music.

The information gathered by sensors and social robot was forwarded to the eWare smartphone app of the informal caregivers and made available to the Tinybot backend. The Tinybot backend used this information to enrich its reasoning about the user’s behavior and construct conversations with the PwD. In case of notifications, the follow-up by the PwD of some of these notifications/reminders is verified based on spoken messages (two-way interaction) to the Tinybot robot, before forwarding these spoken messages or notifications to the involved caregivers. Simultaneously, the information about the interactions between the Tinybot robots and the person with dementia were available to the Sensara backend and used to extend its lifestyle monitoring capabilities.

**Figure 1:**
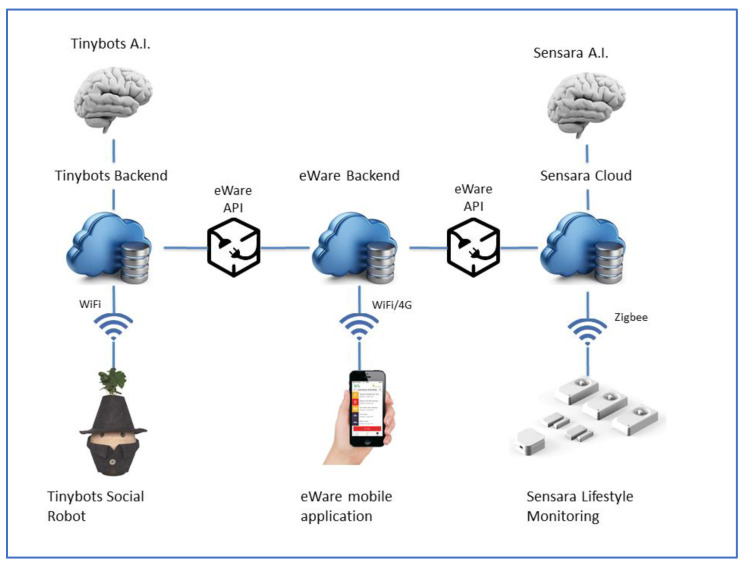
The eWare ecosystem.

In order to connect the devices and the actors in both ways, the eWare ecosystem needs a mobile application and the eWare API. Figure 1 schematizes the descripted framework.

The study has been run during the first lockdown, due to the widespread of the COVID-19 pandemic situation in Italy. It is worth-wile mentioning that the data collection for the final phase of the study was conducted after the end of the isolation of the participants from the social and family life. That represented both a challenge and an opportunity for the researchers: it allowed the understanding of the potential role of the social robotics and lifestyle monitoring technology in a real context of need for assistance and social connectedness, but at the same time it may have caused a negative impact on the assessment of quality of life and stress in the older participants and caregivers, not ascribable to the eWare intervention.

## 2. Methods

In order to collect information on the impact of the system, a field trial was conducted by involving 9 PwD (named from PwD_01 to PwD_09) and their informal caregivers (named from IC_01 to IC_09), in Italy, during October 2019 and May 2020. The study design was divided into three different phases, after the recruitment of the participants: baseline evaluation (T0) at the beginning of the trial, first evaluation (T1) after one month of use, and final evaluation (T2) after 6 months of use.

The recruitment protocol included general information on the subjects, in particular, assessment of the health status and cognitive condition, to check the eligibility of the participants. A team of psychologists selected older people who fulfilled the following inclusion criteria: aged over 65 years old with a diagnosed cognitive impairment from slight to moderate state (ranged from 12–26 at the Montreal Cognitive Assessment, or 18–25 at the Mini Mental State Examination), with Instrumental Activities of Daily Living (IADL) [18] score ≥5, living alone in ordinary housing with a devoted (in)formal caregiver that visited the PwD at least four times per week, able to communicate and interact with the robot, and concurrently not involved in other studies. Exclusion criteria included lack of informed consent from caregiver or PwD, the presence of severe diseases associated with life expectancy less than 6 months (in addition to dementia), or unstable chronic conditions (in either the dementia patient or the family caregiver), and the intention to move to institutionalized care. In total, 9 dyads (composed by the PwD and his/her informal caregiver) were recruited.

Then, the baseline evaluation (T0) consisted in the first real contact with the users and their families, at the start of the field trial. The following scales and tools were administrated during this phase: (a) the Goal Attainment Scale (GAS) [19] was used as primary outcome with the older participants, to set the personal goals to be achieved with the system and evaluate their improvement in the next assessment phases; (b) the Zarit Burden Interview [20] was used to evaluate the reduction or the stability of their burden on the caregivers’ side; (c) the EQ-5D-5L scale [21] was chosen to measure the improvement or the stability of their quality of life in both older participants and informal caregivers. Finally, the attitude towards technology was detected in both the sample during this phase, by using an ad hoc checklist to evaluate the perceived confidence in dealing with specific everyday technologies.

After 4 weeks of use, the first evaluation (T1) aimed at analyzing the usability and the acceptability of the system, with qualitative and quantitative techniques. During this phase, GAS Scale was administrated to the older participants and semi-structured interviews, to detect usability problems were administered to both samples.

The final evaluation (T2) consisted of collecting information about the whole benefits perceived by the users at the end of the test, in order to detect and analyze the impact of the system in the daily life of the older people and their families, as well as to gain knowledge on technology acceptance and usability issues. During this phase, the GAS Scale was administrated to the older people, to collect information on the improvement of the personal goals; the EQ-5D-5L-5L scale was administrated to the caregivers and the older participants, to measure the impact on their quality of life. The Zarit Burden Interview was administrated to assess the burden on the informal caregivers. Finally, in order to determine the acceptability and usability by all the participants, the Questionnaire on Technology Acceptance, based on the Unified Theory of Acceptance and Use of Technology (UTAUT) [22], the System Usability Scale (SUS) [23] and a semi-structured interview were used with the informal caregivers. A semi-structured interview on the same topics was already conducted with the older participants.

The study was approved by the IRCCS INRCA Ethical Board on 18 December 2018 and formalized in 447/DGEN of 31 December 2018. Data collection, usage and storage procedures complied with national laws and the EU’s General Data Protection Regulation (GDPR) including the commitment of participants, the right to access, right to be informed, right to withdraw, and right to data erasure. Data collection was compliant with the principle of data minimization.

### Participants Description

Participants were recruited via public space, personal contacts, and by an IRCCS INRCA team of experts, exploiting collaboration with some branches of IRCCS INRCA (Neurology Unit, Memory Clinic, and Alzheimer’s Assessment Unit) as well as contacts with volunteering associations.

The sample of older people was composed of 8 women and 1 man, with a mean age of 82.56 (±3.54) years old. All of them were widowers, and 6 have reached primary level of education while 3 secondary. The average score of MMSE was 22.11 (±2.69), indeed all the PwD were affected by mild dementia (MMSE between 18 and 24). Regarding quality of life, the mean score of EQ-5D-5L—only Visual Analogue Scale (VAS)—was 70.56 (±20.37), suggesting a high degree of perceived quality of life. Concerning the user’s attitude towards technology, participants were asked to indicate their expertise level in regard to a list of technological devices (Table 1). The user’s rating was given on a 5-point Likert scale from 1 = “I am not able to use this device” to 5 = “I am confident and know how to use it”. An overall low attitude towards technology in older users resulted (M = 1.80; SD = ±1.94). Only the most familiar technologies such as phone, TV and washing machine collected a higher degree of self-perceived attitude.

For what concerns the informal caregivers, the sample is composed of 7 women and 2 men, aged on average 52.78 (±5.99) years old. According to the results, 8 of them were married, while 1 was divorced. Regarding their education, 5 informal caregivers reached tertiary level of education and 4 secondary. Eight of them were still working full time, while one was working part time.

None of the informal caregivers declared to live alone: 7 of them live with their partner and children, whereas 1 lived with the partner only and 1 with their children only. On the attitude towards technology, informal caregivers were asked to rate their skills regarding a list of devices on a 5-point Likert scale, as the older adults. Table 2 shows the results on their digital skills:

The result clearly highlighted an overall very good attitude towards technology (M = 4.39; SD = ±1.43), except for the telemedicine systems.

## 3. Results

Table 3 summarizes the score of all the goals set by the participants at the beginning of the experimentation, to be achieved through the eWare system during the study period.

What emerged from results is a substantial improvement after the first month of use, but the effect did not last throughout the trial as denoted by decreased scores at T2.

PwD_01 did not improve in any settled goals, and worsened in going to the hairstylist because of pandemics. The same for PwD_03 who disconnected and unplugged the system continuously. PwD_02, PwD_04, PwD_05, and PwD_06 improved in most goals after one month, but at the end of the experimentation many scores resulted worsened than the initial ones. Indeed, PwD_04 stated that she listened to Tessa much more before becoming accustomed to its presence. PwD_09 and especially PwD_07 had benefits from eWare system, by considering their GAS scores. Even though the most enhancements are noticeable after T1, great scores were maintained or even increased at T2.

The quality of life, shown in Table 4 and assessed through the VAS scale from EQ-5D-5L at T0, was 71.00 (±19.21). At the end of the experimentation only 5 PwDs were evaluated, and the same indicator resulted in 80 (SD = ±16.73).

The caregiver’s burden was evaluated both before and after the intervention through the ZARIT questionnaire. Table 5 shows the score of the ZARIT for each informal caregiver:

Before the test, it resulted that no caregiver was affected by severe burden; in fact, most caregivers reported mild burden (score between 20 and 40, yellow area), whereas just one participant had little burden (≤20), and two of them reported moderate burden (≥40). The overall situation was confirmed after the intervention, due to the fact that IC_01 reported a lowered burden at T2 whereas IC_05 went from mild to moderate burden through the intervention. Overall, the average stress burden was 34.78 (±12.02) at T0 and it slightly increased to 35.11 (SD = ±10.72) at the end of the experiment, resulting in a substantial stability of this measurement.

Despite the results at ZARIT, which suggests a mild to moderate level of burden, the mean score at EQ-5D-5L scale have revealed a high degree of perceived quality of life. In fact, the mean scores of EQ-5D-5L—VAS scale—was 75.33 (±16.40) at T0 and 66.11 (±23.78) at T2 (Table 6). In this case, a worsening resulted at the end of the experimentation.

The eWare system received a positive evaluation by the informal caregivers in terms of acceptability. In fact, according to the UTAUT scores (Table 7), eWare was easy to use, useful, and adaptive to the needs of the carers. Moreover, the informal caregivers did not feel nervous while using it.

The usability of the system is confirmed by SUS scores [24]. Indeed, 8 out 9 informal caregivers have a score much higher than 68, established as the threshold to define a system usable. Only IC_03 encountered some difficulties during the experimentation (Table 8).

## 4. Discussion

In the next 30 years, the number of older people with dementia will increase significantly, a phenomenon that will negatively impact both the quality of life of the older persons and their caregivers. Therefore, it is becoming increasingly urgent to find solutions that can solve this problem by providing valuable support to both the seniors and caregivers.

The eWare system represents a possible solution to support the older person during his or her daily activities, to help achieve daily goals, and to relieve the caregiver’s stress in caring for a person with dementia.

Despite the small size of the sample, that does not allow a proper generalization of the results, it can be said that the evidence collected may offer relevant aspects to be considered.

First of all, an initial improvement in the perception of personal goals attainment by the older people was observed, by analyzing the positive trend of the self-assessment from T0 to T1. In fact, after one month of use: the majority of the goals (19 out of 34 identified by the sample), were perceived as improved at T1, while 14 received the same evaluation as in T0, and only 1 goal seems not to have improved. Despite this initial positive evaluation, the score for each goal seems to decrease in T2, compared to T1: 17 goals out of 34 were rated as decreased compared to respecting the first evaluation, 14 remained stable between the two assessment phases, while 3 of them were perceived as improved compared to T1.

By analyzing participants’ life events in the period that occurred between the two assessment phases—T1 and T2—, it can be said that an overall negative effect of the psychological, emotional and cognitive status may have been caused by the COVID-19 pandemic. During that period, in fact, the first lockdown and the consequent isolation of the older participants may have caused a general frustration and reluctance in actively participating in the study.

This may partially explain the decrease in the goals’ attainment scores in the second phase. Nevertheless, it is also important to consider some scientific and technological requirements of the technology that may have contributed to the disaffection of the users to the system for pursuing their personal goals in the long-term. Despite the simplicity of the Tinybot interface, which makes it adaptable and easy to use in case of cognitive decline, the system may be lacking what concerns interactive and dialog features. Some studies on social robotics for supporting people with mild cognitive decline and dementia [25,26,27,28,29] have underlined the role of advanced interaction features to engage the users in stimulating activities and social companionship, ranging from voice interaction to tactile feedback. In this case, the simplicity of the task—providing timely reminders—and the relatively easiness of use and appearance of the robot may have been perceived as “not fun” or “not engaging” by the older users in the long-term, who partially lost the interest in using the overall system after the first period. This hypothesis may suggest the need to find a balance between the ease of use of technology and the development of more advanced HRI features, that can attract older users with cognitive decline, without constituting a burden at the same time.

Regarding the caregivers, results, even if not significant, have shown a substantial maintenance of the perceived stress in relation to the caregiving activity at the end of the study. In particular, out of the 9 caregivers recruited, one (IC_01) reported to have significantly decreased his/her perceived stress, while one of them (IC_05) showed an increase. In the other cases, the situation remained stable after six months.

Thus, we can observe a tendentially stable trend in perceived workload, a trend that we still consider positive, if we consider the significant negative impact that the COVID-19 pandemic had on the emotional and psychological level. Furthermore, results have shown an improvement in perceived quality of life. That result may suggest the potential of the eWare system not only in caring for older persons with dementia, but also in supporting some aspects of the caregivers’ lives, contributing to a higher quality of life. However, the involvement of a larger sample is needed to confirm this preliminary observed tendency.

Another factor that needs to be taken into consideration is the fact that dementia, being a degenerative disease, requires increasing care and assistance over time, consequently causing increasing stress and workload [30]. Therefore, the fact that the eWare system enabled caregivers to keep their perception of stress and workload stable for the duration of the trial is an important positive aspect to take into consideration. In particular, the opportunity of receiving information on the progress of the dementia, seems to improve the sense of control over the caring situation and the perception of security [31].

Finally, regarding the acceptability of the system collected through the UTAUT questionnaires, the caregivers described the system as pleasant and its usability as good.

The system is indeed simple and intuitive, so the fact that users did not detect any usability problems is in line with what we expected. Nevertheless, the results obtained from the older participants show that the system should probably be enriched and expanded with other features, which can stimulate older people more, giving them the opportunity to interface with a product that actually fits their needs and is superior to similar systems already available on the market.

## 5. Conclusions

The eWare system seems to have good potential, but modifications will have to be made in order to meet the needs and especially the difficulties of a person with dementia, which are different from the difficulties of a healthy older person. Indeed, it should be remembered that when dealing with dementia, the normal difficulties associated with healthy aging are bound up with the specific difficulties of that type of condition.

In addition, dementia can manifest in different ways from person to person, making it even more important to understand what the individual’s real needs and challenges are.

Despite this, results demonstrated the potential of social robotics and lifestyle monitoring, in line with the literature in the field. In fact, thanks to such technological ecosystem, the caregiver can monitor the older person’s behavior and recognize any unusual or worrisome behavior, without necessarily being at home with them, thus allowing decisions on how to manage the time and care to be devoted to the older person.

## Figures and Tables

**Table 1:** Attitude towards technology in older users.

Item	Mean	SD
Phone	4.88	0.33
Mobile phone	3.86	0.99
Smart phone	2.00	1.41
TV/Remote control	4.89	0.31
CD/DVD player	0	0
PC	0	0
Internet	0	0
Tablet	0	0
Bank ATM	2.40	1.74
Devices to increase your safety (pressure measurer, automatic device to open/close the door, reclining chair, emergency alarm …)	3.33	1.80
Washing machine	3.78	1.75

Mean = mean value; SD = Standard deviation.

**Table 2:** Attitude toward technology in informal caregivers.

Item	Mean	SD
Phone	5	0
Mobile phone	5	0
Smart phone	4.89	0.33
TV/Remote control	5	0
CD/DVD player	5	0
PC	4.89	0.33
Internet	4.89	0.33
Tablet	4.88	0.35
Bank ATM	5	0
Devices to increase your safety (pressure measurer, automatic device to open/close the door, reclining chair, emergency alarm …)	4.86	0.38
Washing machine	5	0
Telemedicine	2	0
Alarm system	5	0
Other	0	0

Mean = mean value; SD = Standard deviation.

**Table 3:** GAS scores at the beginning, one month later, and at the end of the test.

	Goal(s)	GAS (T0)	GAS (T1)	GAS (T2)
PwD_01	Remind to have breakfast on time	−2	−2	−2
	Remind to have dinner on time	−2	−2	−2
	Remind to wait for taking a nap	−1	−1	−2
	Remind to do physical activity at home	−2	−2	−2
	Take care of the cat	0	0	−2
	Remind to go to the hairdresser once a week	0	0	−2
PwD_02	Remind to call the sons	−2	−1	−1
	Remind to close front door	−1	0	−2
	Remind to close the cookers	−1	0	−2
	Remind to go to the hospital	−2	0	−2
PwD_03	Remind to have lunch on time	−2	−2	−2
	Remind to feed the dog	−2	−2	−2
	Remind to have dinner on time	−2	−2	−2
	Remind to take medicine	−2	−2	−2
PwD_04	“Good morning” message	−2	0	−2
	Remind to have breakfast on time	−1	0	−2
	Remind to iron or hang out the clothes	−2	−1	−2
	Remind to take medicine	−1	+2	+1
PwD_05	Remind to go to the hospital	−2	0	−2
	Remind the arrival of the housekeeper	−2	−1	−2
	Remind to have lunch on time	−2	−2	−2
PwD_06	Remind to have breakfast on time	−1	0	−1
	Remind to change medical patch	−2	−1	−2
	Try to remember the day of the week	−1	0	−1
	Remind to have dinner on time	−1	0	−1
PwD_07	Remind to have breakfast on time	−1	0	0
	Remind to take medicine	−2	0	0
	Remind to drink water	−1	−2	0
	Remind to walk	−2	−1	−1
PwD_09	Remind to have breakfast on time	−2	−2	0
	Remind to have lunch on time	−2	−2	−2
	Remind to take medicine	−2	+2	0
	Remind to have dinner on time	−2	−2	−2
	Remind to close the house door	−1	0	+1

PwD = Patient with dementia (named fron PwD_01 to PwD_09); T0 = baseline evaluation; T1 = evaluation after one month T2 = final evaluation.

**Table 4:** VAS scores of PwD at the beginning and at the end of the test.

	PwD_01	PwD_02	PwD_03	PwD_04	PwD_05	PwD_06	PwD_07	PwD_08	PwD_09
**VAS (T0)**	65	86	85	44	100	52	53	58	92
**VAS (T2)**	-	100	-	100	-	60	70	-	70

PwD = Patient with dementia (named fron PwD_01 to PwD_09); VAS = Visual Analogue Scale; T0 = baseline evaluation; T2 = final evaluation.

**Table 5:** ZARIT scores of informal caregivers at the beginning and at the end of the test.

	IC_01	IC_02	IC_03	IC_04	IC_05	IC_06	IC_07	IC_08	IC_09
**ZARIT (T0)**	49	38	45	39	34	7	30	32	39
**ZARIT (T2)**	26	37	57	27	42	20	37	33	37

IC = Informal Caregiver (named from IC_01 to IC_09); T0 = baseline evaluation; T2 = final evaluation.

**Table 6:** VAS scores of informal caregivers at the beginning and at the end of the test.

	IC_01	IC_02	IC_03	IC_04	IC_05	IC_06	IC_07	IC_08	IC_09
**VAS (T0)**	96	50	70	78	99	74	50	75	86
**VAS (T2)**	90	10	75	70	40	80	80	70	80

IC = Informal Caregiver (named from IC_01 to IC_09); VAS = Visual Analogue Scale; T0 = baseline evaluation; T2 = final evaluation.

**Table 7:** UTAUT scales scores of informal caregivers at the end of the test.

Scale	IC_01	IC_02	IC_03	IC_04	IC_05	IC_06	IC_07	IC_08	IC_09	Mean
**ANX**	1.0	1.0	1.0	1.0	1.0	1.0	1.0	1.0	1.0	1.0
**ATT**	3.3	5.0	3.7	4.3	3.3	4.0	3.3	4.0	4.3	3.9
**FC**	5.0	3.5	5.0	5.0	5.0	5.0	5.0	5.0	5.0	4.8
**PAD**	4.7	5.0	4.0	5.0	3.3	5.0	3.7	4.7	5.0	4.5
**PENJ**	3.4	3.6	2.8	3.8	2.0	3.4	2.6	2.2	3.4	3.0
**PEOU**	5.0	4.4	3.6	5.0	5.0	5.0	5.0	5.0	5.0	4.7
**PU**	5.0	4.3	4.0	5.0	2.7	4.7	3.3	3.7	5.0	4.2
**Trust**	5.0	5.0	4.0	5.0	5.0	5.0	5.0	4.0	4.0	4.7

Scale from 1 to 5. ANX: Anxiety, ATT: Attitude, FC: Facilitating Conditions, PAD: Perceived Adaptability, PENJ: Perceived Enjoyment, PEOU: Perceived Ease of Use, PU: Perceived Usefulness; IC = Informal Caregiver (named from IC_01 to IC_09).

**Table 8:** SUS scores of informal caregivers ar the end of the test.

	IC_01	IC_02	IC_03	IC_04	IC_05	IC_06	IC_07	IC_08	IC_09
**SUS SCORE**	100	97.5	52.5	97.5	92.5	97.5	95	90	100

SUS = System Usability Scale; IC = Informal Caregiver (named from IC_01 to IC_09).

## Data Availability

The datasets generated, used and analyzed during the trial and its preceding pilot trial are or will be available from the corresponding author upon reasonable request.
